# “Be Kind But Not Too Kind”: Black Males' Prosocial Behaviors in the Face of Dehumanization

**DOI:** 10.1111/jora.12746

**Published:** 2022-04-05

**Authors:** Johari Harris, Ann Cale Kruger

**Affiliations:** ^1^ University of Virginia; ^2^ Georgia State University

**Keywords:** adolescent Black males, anti‐Blackness, prosociality, identity

## Abstract

This qualitative study examined how adolescent Black males (*N* = 12) talk about their identities, prosocial behaviors, and connections between them. Of special interest was whether and how the participants included their experiences of dehumanization. Focus group data were analyzed using modified analytic induction. Participants felt good about their racially gendered identities but felt they occupied a precarious position in the United States. Participants’ beliefs about how others viewed them motivated restraint from engaging in too many prosocial acts to prevent appearing vulnerable. Participants explicitly referred to their experience of oppression in these discussions and its interaction with identity and prosociality. Results suggest research must consider how macro‐level processes like racism influence the identities and prosocial behaviors of adolescent Black males.

During adolescence, Black males in the United States face complex and daunting challenges (Brooms, 2020; Brooms & Perry, 2016). In K‐12 schools, common discipline practices result in the suspension and expulsion of Black boys at higher rates than female and White peers (Heilbrun et al., 2018) and contribute to their marginalization. In the community adolescent Black males are heavily policed and significantly more likely than peers to be incarcerated (Anderson, 2000; Jones, 2014). These challenges arise from the systemic dehumanization (Way & Rogers, 2017) of Black boys and men in the United States and reflect the anti‐Blackness (Dumas, 2016) that is foundational to and endemic within the culture.

Despite growing up in the context of anti‐Blackness, many Black youth develop positive racial‐ethnic identities (REI); these youth have higher self‐esteem, better short‐ and long‐term academic outcomes, and are less affected by discrimination than peers who have more negative feelings about their REI (Chavous et al., 2003; Jones & Neblett, 2016; Mandara et al., 2009). Less research has examined the relationship between REI and behavioral outcomes, and the research that has been done has often focused on anti‐social and risk‐taking behaviors (e.g., fighting and sexual activity) and until recently overlooked REI’s possible relationship to prosocial behaviors.

Prosocial behaviors are voluntary actions that benefit another (e.g., helping and sharing), and they contribute to academic and social success, two important outcomes of adolescent development (Flynn et al., 2015; Hart & Fegley, 1995). Adolescents are increasingly likely to engage in prosocial behaviors (Barr & Higgins‐D’Alessandro, 2009; Fabes et al., 1999; Kanacri et al., 2014), and this is influenced by both external (nationality, school, and racial‐ethnic background) and internal (perspective‐taking and empathy) factors (Fabes et al., 1999). There is an emerging research literature on the promotive effects of REI on prosocial behaviors among Black youth. Since race *and* gender are highly salient in adolescents’ experiences and affect psychological outcomes (Rogers et al., 2015), a refined intersectional framing of identity merits attention here. In the present study we explored how groups of adolescent Black males discussed their racially gendered identities and their prosocial behaviors. We paid special attention to any links they made between their identity and prosociality and to whether and how their experiences of systemic oppression and dehumanization were included in these conversations.

## The context of development

Anti‐Blackness theorists assert Black people are positioned as other than human, and this provides justification for others to deny them basic human rights. Dumas (2016) writes, “there is a concern with what it means to have one’s very existence as Black constructed as problem—for White people, for the public (good), for the nation‐state” (pp. 12). Merely existing as Black presents a level of risk. Adams‐Wiggins and Taylor‐García (2020) contend that while it is valuable to generally consider contextual factors in development, one must explicitly critique and address the ubiquity of anti‐Blackness.

Historical and contemporary White fear of Black males has created oppressive social and economic structures (i.e., legal systems, schools, political systems) to control their bodies (Coates, 2017; Ferguson, 2001; Howard et al., 2012). The high rates of interpersonal and community violence that disproportionally affect young Black males are, in part, a result of the devaluation of their lives (Leovy, 2015). For example, proactive policing policies often shape the nascent identities of adolescent Black males as they may come to conflate power and respect with aggression and force (Jones, 2014). In addition to disproportionate discipline, young Black males receive more placements in behavioral or special education programs (Vincent et al., 2012) and are more likely to attend underfunded schools and have under‐qualified teachers, two factors critical to school success (Darling‐Hammond, 2004). Despite protective factors, such as strong family ties, this level of systemic bias, rooted in anti‐Blackness, and present across so many sectors of their lives renders them particularly vulnerable and can shape their self‐perceptions and their later performances of what it means to be an adolescent Black male in the United States (Cassidy & Stevenson, 2005; Rogers & Way, 2016).

## Phenomenological variant of ecological systems theory

The phenomenological variant of ecological systems theory (P‐VEST) is an identity‐centered ecological model that focuses on an individual’s *perceptions* of their macro‐ and micro‐social contexts (Spencer, 2006, 2008). Rather than positioning an adolescent as a passive participant being “acted upon,” in P‐VEST the adolescent actively makes meaning of daily experiences, and this meaning influences their identity and behavior during development and across contexts. The judgements on *how* they are viewed by others, also known as reflected appraisals, are a key part of the developmental process as adolescents may integrate certain aspects of these appraisals into their burgeoning identities.

Phenomenological variant of ecological systems theory is comprised of five bidirectional components: emergent identity, net vulnerability, net stress engagement level, reactive coping mechanisms, and stage‐specific outcomes (Spencer, 2006, 2008). An individual’s racial‐ethnic and gender identities are examples of emergent identities. Emergent identities represent the way people see themselves *across* contexts. Net vulnerability balances possible risk factors (e.g., low SES and high‐crime communities) with possible protective factors (supportive parents and strong community ties) to determine overall risk. Similarly, net‐stress engagement balances an adolescent’s actual engagement with stress with their reactive coping mechanisms. The adolescent’s net engagement with stress will lead to positive or negative outcomes that are represented within the stage‐specific outcome component. We focus here on three components of P‐VEST: net vulnerability, emergent identities, and stage‐specific outcomes. We foreground anti‐Blackness as a contributor to net vulnerability and examine whether and how adolescent Black males speak about their vulnerability in relation to their emergent identities and to the stage‐specific outcome of prosociality.

## Intersectionality

Intersectionality theory describes the ways interlocking systems of oppression shape one’s experience (Cho et al., 2013). Rather than viewing socially mediated identities as independent and value‐neutral, an intersectional lens understands “Categories such as race, gender, social class, and sexuality do not simply describe groups that may be different or similar; they encapsulate historical and continuing relations of political, material, and social inequality and stigma” (Cole, 2009, p. 173).

In her seminal work on intersectionality theory, Crenshaw (1991) showed that systemic oppression by race, gender, and citizenship status is compounded in an individual’s experience by highlighting the different outcomes for Black women, White women, and immigrant women victims of sexual violence. Intersectionality theory also informs research on those who simultaneously hold privileged and disadvantaged identities, such as Black males. Their gender affords them a level of privilege in the patriarchal U.S. culture, while they are continuously oppressed by its virulent strain of racism. While maintaining a thorough appreciation for context and associated challenges for adolescent Black males, using an intersectional approach to study developmental outcomes is critical to deepen our understanding (Gaylord‐Harden et al., 2018).

## Emergent identities and prosocial behaviors

In 2006 McMahon and others showed that among Black adolescents from low‐income backgrounds, empathy predicted prosocial behavior. Unlike earlier research (Eisenberg et al., 1991) on children in general, in McMahon’s study Black boys had higher scores on empathy than Black girls, suggesting that Black children’s development cannot be understood through literature on White or other children and must be studied through an intersectional lens. A largely quantitative literature exploring the relationship between REI and prosocial attitudes and behaviors has begun to emerge. An early study (Jagers & Mock, 1993) suggested a positive relationship between Black adolescents’ REI (measured as an endorsement of cultural values) and prosocial attitudes. Similarly, in an ethnically diverse group of adolescents, Smith et al. (1999) identified a path linking self‐esteem directly and indirectly to prosocial attitudes; to a lesser degree ethnic identity predicted prosocial attitudes mediated by self‐efficacy.

In a hierarchical cluster analysis Belgrave et al. (2011) measured ethnic identity, empathy, anger management, and beliefs about aggression to predict aggressive and prosocial behavior among Black adolescents. They identified four profiles each for adolescent Black boys and Black girls and showed that for Black boys a positive ethnic identity was related to lower aggression and higher prosociality, suggesting that REI has protective and promotive effects on their development.

More recent research on REI and prosociality in Black youth has considered the function of discrimination and oppression in identity development (e.g., Lozada et al., 2017) and the resulting motivation to engage in prosocial activity to advance social justice (e.g., Wray‐Lake & Abrams, 2020). In studies of Black college students, White‐Johnson showed that experiences of discrimination along with positive White‐Johnson, 2012, 2015) and parental preparation for bias socialization messages (2015) predicted prosocial attitudes and behaviors contributing to the Black community. Maiya et al. (2021) demonstrated that parental racial‐ethnic socialization messages contributed to the prosocial attitudes of Black college students, mediated by their REI and religious identity. The authors interpreted their measure of prosocial attitudes as representing respectable citizenship and argue that it is motivated by the desire to counter negative stereotypes.

Of greatest relevance to the present study, Lozada et al. (2017) conducted a large study of adolescent Black males’ sociopolitical development and its relation to prosocial behaviors. The authors defined the participants’ “oppression analysis” as a latent variable that emerges from the centrality and positivity of their REI along with their identity as a member of an oppressed minority, comparable to the development of critical race consciousness and the understanding that racism is used to maintain White privilege. They found that parental socialization messages of racial pride, but not experiences of discrimination at school, predicted oppression analysis in the participants. Oppression analysis in turn directly predicted social emotional skills and directly and indirectly (through social skills) predicted prosocial behaviors. Further, prosocial behaviors were directly and independently predicted by experiences of discrimination at school. Although this model held for Black boys at predominately White schools as well as at predominately Black schools, boys at predominately White schools scored higher on the variables contributing to oppression analysis, suggesting that experiences with minority status at school may prompt critical consciousness. Older boys were more likely than younger boys to identify as an oppressed minority, suggesting that the development of social cognition and/or the accrual of experiences contribute to this.

Lozada and colleagues measured prosociality in this study as endorsing behaviors such as helping, comforting, and donating. They suggested that prosociality can be motivated by the development of a critical social analysis and independently by experiences of oppression. It remains to be investigated how helping others in early to mid‐adolescence (Lozada et al., 2017) develops into civic engagement and advancement of social justice in college students (Mayia et al., 2021; White‐Johnson, 2012, 2015).

Quantitative research has identified REI as a mediator between social experiences and prosocial outcomes (Belgrave et al., 2011; Fergus et al., 2005; Harris & Kruger, 2019; Lozada et al., 2017; Maiya et al., 2021; Quimby et al., 2018; Smith et al., 1999; White‐Johnson, 2012, 2015). It should be noted that others have investigated REI as a moderator (e.g., Fisher et al., 2020) and as an outcome variable (e.g., DiClemente et al., 2018). There has been scant qualitative study of REI and prosociality in Black adolescents (but see Deutsch, 2005). Questions remain about how Black youth conceptualize and discuss their experiences, their identity, and their prosociality. The current study adds to the existing quantitative literature with a qualitative study of Black adolescent boys’ discussions of these matters.

## Current Study

We conducted an earlier (explanatory sequential mixed method) investigation to examine if and how adolescent Black males’ racial‐ethnic and gender identities predict self‐reported prosocial behaviors within an urban school context (Harris & Kruger, 2019). Quantitative results indicated that racial public regard (how a participant thinks others feel about the participant’s race) positively predicted prosocial behaviors. In addition, gender private regard (how participants feel about their own gender) and gender public regard (how others feel about the participant’s gender) independently predicted prosocial behaviors (Harris & Kruger, 2019).

It remains an empirical question *how* racial public regard and gender private and public regard specifically or identity generally are related to prosocial behaviors and whether and how the hostile context of anti‐Blackness influences these developmental processes. Here we use qualitative data drawn from the previous study to investigate how the participants discuss these matters in the developmentally salient context of a peer group. There were two focus groups composed of those who scored at the lowest and highest levels, respectively, on an instrument measuring self‐reported prosocial behavior. Participants were not aware of this grouping strategy or of their scores on the measures. Informed by P‐VEST, we assumed that listening to discussions among adolescent Black males would provide some access to their meaning‐making and possibly begin to explicate the relationships among these variables. Building on previous findings specifically addressing adolescent Black boys’ prosociality (Belgrave et al., 2011; Harris & Kruger, 2019; Lozada et al., 2017), we explored the following questions.

1. When prompted, how do adolescent Black boys discuss identity, discrimination, and prosociality? Do the high and low prosocial groups vary?
2. Do participants’ discussions specify connections between the concepts? Do the groups vary?

## METHODS

### Participants

All participants (*N* = 12) identified as male and as Black or African American. The sample size constitutes all available participants for the qualitative portion of the larger mixed‐method study. The mean of prosocial scores on the Prosocial Behavior Scale (Solomon et al., 2000) for the total sample (*N* = 131) was 3.04. The participants invited to participate in the qualitative portion scored in the top and bottom quartile of prosocial scores and represented over 50% of their group’s respective quartile, supporting saturation efforts (Mason, 2010). See Table 1 for details about the composition of the two groups.

**Table 1:** Age, Identity Scores, and Prosocial Scores in Two Focus Groups

Variable *Ms*	Low Prosocial Group	High Prosocial Group
Age	14.8	15.2
Prosocial behaviors	1.8	4.6
Gender centrality	3.6	3.7
Gender private regard	4.6	5.0
Gender public regard	3.5	4.3
Racial centrality	3.5	4.3
Racial private regard	3.9	4.3
Racial public regard	2.3	3.7

The two groups met separately; there were six participants in each group. All participants attended College for All (CA) (a pseudonym), a public charter high school operating within a large metropolitan school district in the southeastern region of the United States. Over 98% of students attending CA identify as Black or African American. College for All is a Title 1 school (a federal designation for schools with more than 40% of the students living at or below the poverty line) located in an economically depressed area. Additionally, all students attending CA qualified for the federal lunch subsidy, indicating their family incomes were at or below 130% of the national poverty level.

### Procedures

The IRB at Georgia State University approved the present study (IRB00000716). All invited participants elected to participate in a focus group. They were provided a pizza lunch and 10 dollars for their participation. Focus groups were conducted on school grounds in Fall 2017. Each session took approximately 65 min, and both were audio‐recorded. See Appendix A for the focus group questions. Recordings were later transcribed for analysis. The first author acted as the facilitator and note taker for both groups, which are referred to here as low and high prosocial groups.

### Focus Groups

Focus groups provide a space for participants to make meaning of their experiences though dialogue with those who share cultural experiences (Parker et al., 2012). Information gleaned from the dialogue *between* participants is equally important to information told directly to the researcher (Ivanoff & Hultberg, 2006). Thus, the focus groups in the present study were the unit of interest, not the individuals themselves, and ideas that emerged from the focus groups were viewed as collectively constructed (Smithson, 2000). Research has noted that focus groups, unlike one‐on‐one interviews, create spaces where adolescents feel more comfortable to share experiences and less compelled to provide answers they believe the facilitator may want (Peterson‐Sweeney, 2005). Moreover, given the importance of peers during this developmental period, focus groups may provide insight into how meaning is co‐constructed in this highly salient social context. While the small focus groups of the present study preclude generalizability, they provide access to how adolescent Black males reporting high or low levels of prosociality discuss the relevant matters.

### Analysis

Findings were interpreted through the lens of P‐VEST and Intersectionality. P‐VEST guided interpretation by examining how emergent identities may affect and be affected by net vulnerability and how that, in turn, is related to behaviors. By drawing upon intersectionality theory, issues of race‐ethnicity, gender, and power are not adjacent but are central to understanding participants’ experiences.

#### Modified analytic induction

We used modified analytic induction when coding qualitative data (Creswell & Clark, 2017). In modified analytic induction, prior findings and/or hypotheses are used to create codes (Suizzo et al., 2016). Findings from the previous quantitative study informed the focus group questions and the coding process of the present study (see Appendix B for details on the measures). Specifically, constructs measured quantitatively (*racial private regard, racial centrality, racial public regard, gender private regard, gender centrality, gender public regard, prosocial behaviors*) and the contextual variable *perceptions of discrimination* were used as initial descriptive codes and were applied line by line to the transcripts. Any lines containing irrelevant information (such as discussing favorite pizza toppings) were excluded from coding.

Next, the lines coded with a specific descriptive code were reviewed together to create interpretive codes that added a layer of specificity. For example, on lines coded as prosociality (a descriptive code), more specific interpretive codes were added. A line such as “I bring her her favorite snack” was further coded with the interpretive code *acts of generosity,* as it represented a specific type of prosociality, a generous act towards peers. This process was repeated for each descriptive code. Last, the interpretive codes were reviewed, and emerging themes identified. For example, interpretive codes *respect*, *acts of generosity*, and *deference* contributed to the theme *prosociality* as these interpretive codes were related to how the participants constructed this larger idea. Table 2 reflects the codes and themes developed.

**Table 2:** Qualitative Codes

Descriptive Codes	Interpretive Codes	Themes
Racial public regard	Intersectionality Community Feeling bad/good about race	Emerging identities and dehumanization
Racial private regard		
Racial centrality		
Gender public regard	Feeling good about gender Individuality	
Gender private regard		
Gender centrality		
Prosocial behavior	Kindness Respect Deference Generosity	Prosociality and meaning making
Experiences of discrimination	Negative experiences	Dehumanization

Data were coded with an educational psychology doctoral student using the mixed method coding software Dedoose (Dedoose Version 2016). Prior to independent coding, we discussed the research questions to ensure shared understanding of the descriptive codes and their relation to the constructs of interest. Next, we independently coded the transcript from one focus group, attending only to descriptive codes. Dedoose was utilized to calculate interrater reliability (Kappa = .84) as well as maintain the codebook. Next, we independently coded both focus group transcripts with descriptive codes and then interpretive codes. Upon completion of coding, we came together to review codes and determine themes which linked interpretive codes together. If differences between interpretive codes emerged, we discussed them and came to an agreement about the best code for any line. Themes were generated through consensus. In the following results, only pseudonyms are used.

#### Researcher Positionality

The first author is a Black woman who centers her research how contexts of development (e.g., schools, communities) can be spaces of liberation rather than contexts of oppression. She believes that we must listen to the voices of Black youth to best understand their development and resistance to the anti‐Blackness endemic to the U.S. context to achieve this and therefore uses research methodology that centers participants’ perspectives. The second author is a White female senior faculty member in a large urban research university in the Southeastern US. During the last 15 years her research has focused on Black adolescents’ girls’ thoughts and feelings related to psychological development within urban school contexts. The first author conducted focus groups and led analysis of the data.

The positionality of the authors informed the research methodology. Given the authors positionality as women, it was particularly important to utilize methodological approach that centered the voices of participants, adolescent Black males. Through modified analytic induction and focus groups, participants’ beliefs were the guiding point for focus group questions and follow up questions. Moreover, the subsequent analysis used a lens that centered participants’ race and gender through a macro‐level lens to understand their experiences.

## RESULTS

### Theme 1. Emerging Identities: “Blessed” and “Highly favored”

Participants described their feelings about their gender identity, REI, and racially gendered identity. In both groups the words they used to describe their gender were: “powerful,” “trustworthy,” “strong and smart,” and “leader.” For race, participants said “scared,” “discriminated,” “unique,” “targeted,” “humble,” “powerful,” “blessed,” “talented,” “highly favored,” and “feared.” When asked about what words come to mind regarding their racially gendered identity as adolescent Black men, the following descriptions emerged: “fear,” “oppressed,” “flashy,” and “provider.”

We asked participants if their perspectives represented their point of view as a Black person, a male, or a Black male. All participants stated their perspective was of a Black male. As Rico stated, the identities “come together.” Both groups agreed they could not untangle gender from race. Interestingly, despite stating the two identities were intertwined, the conversations in the groups focused primarily on REI.

Participants expressed high private regard for their REI. In both focus groups, participants discussed that despite Black Americans facing a range of historical and present‐day traumas, they thrive and maintain a strong sense of community. Some went further and cited Black Americans’ resilience as evidence of “Black people being blessed” and a source of pride for them. Tyrese extended this sentiment.

Yeah, I was going to say, I feel good about being Black because it just—I feel like I’m bonded with everybody. There’ll be people I don’t even know. I can just be like ‘Hey, how you doing?” or “What’s up?’

### Theme 2. Experiences of Dehumanization: “As I Became Bigger, I Became a Bigger Target”

Participants’ responses on how other people viewed their race (racial public regard) were mainly negative. They were keenly aware of the discrimination Black Americans faced. Zaire from the low prosocial group used the following example of segregated communities and media portrayals of Black Americans to illustrate this point.

Because you can see, in everything we do, we already got some hate thrown at us. Like most of the movies and shows on TV, it’s White folks on there. Then once you go into the suburbs—cuz they separate it, the suburbs, then urban. All the Black folks in the urban with all the broke down stuff. Messed up TVs, all that. Then you got the suburbs with the White folk.

While he spoke, other participants nodded their heads, quietly affirming his frustrations about the persistent inequality in America. Participants in both groups used words like “dangerous,” “feared,” and “targeted” when describing other peoples’ feelings and opinions about Black people and Black males. When probed on the fear aspect, Chad from the high prosocial group had this to say:

…I say feared…cuz I know I’ve been walking down the street before and a cop just looked at me and approached me for—and talked to me, stuff like that. I’ve been in places where White people see us, and they clutch their purses. They look at us the wrong way, go to the opposite side of the way.

They believed others viewed their racially gendered identity in a positive light only when related to certain stereotypes about Black men. In both groups, participants stated the two areas in which they were positively perceived by others were in sports and with women. To quote Rico in the high prosocial group, “They like us when it comes to our athletic abilities and sexual desires.” In the low prosocial group, Ray said,

They pitch to us, like when we leave high school, sports are gonna get us out. They don’t pitch us as doctors or lawyers or stuff like that. They try to teach us to dumb us down, so that—because the Black man in power is—he’s dangerous to everybody else… I think they only accept us when we’re making their money instead of takin’ away from their pockets. That’s when they try to get rid of us.

Regarding sex, the commentary moved into a back and forth about White girls approaching them, saying things such as, “We like Black boys.” Participants were not disturbed by these experiences but rather amused and a bit boastful. For example, when Michael from the high prosocial group proudly stated, “White girls love us,” all other participants vigorously agreed.

Participants were then asked to think deeply about instances when they were *treated* a particular way because of their race. Most examples provided were of other people treating them with suspicion. Participants surmised it was due to their race or their positioning as Black males. Tyrese shared the following experience,

…I was on the train. A couple players on my football team, we were just walking on a train. People were giving us looks. We were just chilling. Some people would like to move away. I don’t know why. I guess cause we were Black.

#### Differences between high and low prosocial groups

When asked to share specific experiences of being treated a certain way due to their identity, the low prosocial group recounted only examples of negative treatment. During their conversations and descriptions of events, low prosocial group members frequently used the term *they*. When probed on whom *they* referred to, Maurice stated “White people” absentmindedly while playing with his pencil, and the others quickly nodded in agreement. When asked to elaborate, Maurice made eye contact and firmly stated, "Look at our president^1^. He not here for us…He try and take everything Obama made to help us out…He's not for us.” Zaire quickly agreed and referenced the recent gentrification of their urban neighborhood as evidence of the contentious relationship between Black and White Americans. Zion put it in starker terms and proclaimed, “It’s about like Black versus Whites. It's like a war between us to who's better or who can get the job done.”

The group went on to state these opinions were formed as they “got bigger” with Ashad elaborating, “As I got bigger—I became a bigger target.” The justice system also influenced these views; Maurice quietly stated, “The White man have justice over *us*.” Others in the low prosocial group stated learning about American history shaped their current perspective. Ray explained, “It started when like ever since I started learning about slavery… As I got older, as I started to notice history repeats itself. It gained on me. It got bigger.” Participants agreed learning more about the world and their positions as young Black males soured them to the intentions of White Americans.

The high prosocial group presented a different perspective on the relationship between White and Black Americans. When asked to share experiences of positive or negative treatment predicated on race, only half of the group reported negative experiences they believed to be due to their race. This high prosocial group also used the word *they* frequently but when probed further about it, participants stated “they” referred to anybody. Tyrese explained:

I see it everybody the same. It’s just that’s what they see, what they perceive the world as and what you see it as may be different because so many people see the world in their own type of way.

Chad expounded on Tyrese’s point, explaining:

Some people, and I’m not even gonna label them as White people, just they cool people, I’m not gonna be like, ‘Oh that’s a White person over there.’ I’m trying to get past that. I still wouldn’t tolerate a White person calling me the n‐word tho.

Participants in both groups stated that others’ feelings about Black Americans did not affect how the participants in turn behaved toward them. However, the low prosocial group was more explicit than the high prosocial group in naming and critiquing White people.

### Theme 3. Prosociality: “If You Need Help, I Got You.” “Don’t Mean We Cool, But I Respect You”

To probe their thinking about prosociality in a conversational way, participants were first asked to provide examples of kindness (The question to probe prosocial behaviors was framed around the word “kindness” due to the lack of conversational use of the word prosocial). The examples were, by and large, acts of generosity. The two most common acts were sharing food or money with those around them. Responses included “Help with work and stuff,” “Kind is when you nice to everybody, and you just—you just not mean to nobody,” “Helping with priceless things. Help with work and stuff,” and “You just nice to everybody. Just a good person.” When further probed, participants agreed kindness was going beyond what was expected of you. Jared and Kenny in the high prosocial group discussed how they were kind to other students at their school. Jared said, “Just on a day‐to day‐basis, opening the door for anybody‐ just hold the door.” Kenny stated, “Matter afact, just earlier I just gave someone a dollar because he asked me for a dollar…if you need help, I got you.”

Besides generosity, respect was one of the first words participants in both groups used when asked to define being kind to another person. When asked to expand upon respect, participants stated being respectful was different from being kind or generous. According to participants, one is often prompted to engage in prosocial behaviors with another person *because* of respect for them. Participants defined respect as an understanding of physical and emotional boundaries. As Kenny succinctly put it, "Respect is people knowing what I tolerate and what I don't tolerate." Kindness, or even liking another person, was not required to give or receive respect. Bobby shared:

Like, I don't like you, but I respect you as a man. You see someone getting picked on, or you see‐ if you see an incident going on…like a fight. The person he got a big presence and like, ‘Nah man I ain’t gonna fight you.’ I respect you. Don't mean we tight. That don't mean we cool, but I respect you for being a bigger person…I respect you as a man, but that don't mean we got any type of friendship, or anything. I just respect you.

Within their school context, participants showed respect to peers and school staff through acts of deference. As an example, participants cited acquiescing to a teacher’s demand without complaint or comment, even if they did not understand or agree with the request. Zion stated, “Respect is like if she tell you to be quiet, just be quiet. Don’t talk back.” Similarly, with peers, participants showed other students respect by not engaging in or escalating a potentially volatile situation. When asked about which they valued more, respect or kindness, all participants stated respect. Participants felt it was essential to be respected across contexts. They stressed a nice person who did not show them respect was essentially worthless.

### Theme 4. Meaning Making: “Be Kind but Not Too Kind”

Participants reflected on how their identity, the context of dehumanization, and their prosocial attitudes interact in their lives. They described how racial socialization influenced their identity and that their identity was influential in how they treated others. Some participants said they were raised to be kind, and Black people in general are community oriented. When asked how their REI motivated them to be kind to others, Tyrese and Tommy in the high prosocial group explained the relationship. Tyrese started by stating, “It’s in my heart,” and Tommy confirmed these sentiments in saying, “It’s just the way we were brought up.” The low‐prosocial group had similar beliefs, and Locksley from the group discussed the community aspect that was important to his identity.

I don’t wanna be—I don’t wanna come out the hood doing good and not leavin’ them. It’s like, say I did come out, I make it, and then like, dang, he was over here and he ain’t even doin’ nothin’ for us. I wanna at least give back so they could have an idea like do right.

Although motivated to give back to their own community, the threat of being stereotyped in the larger world was simultaneously looming. To counter negative stereotypes about Black males being dangerous, some participants were motivated to be successful or kind to show the person the error of their ways. Zion and Ashad from the low prosocial group described a hypothetical situation with a woman who may lock her doors upon sighting a young Black male.

Zion: Okay…so that moment you walk by the car, and she lock the doors so you—that makes you wanna go harder so 10 years later you a successful Black man. She lookin at the TV. She see your name pop up and she say, ‘Oh that look exactly like the Black boy that walked by my car and I locked the door on him.’

Ashad: I think if they lock the doors, the next day come say something to her. Say something nice to her. Don’t try to act aggressively and keep doing that. Then eventually, she’ll feel stupid for even trying to treat you like a threat or something.

#### Differences between high and low prosocial group

The participants with the highest prosociality scores were explicit about the limits and risks of being kind, about needing to protect themselves from appearing too kind and thus being vulnerable. This was not observed in the low prosocial group.

When asked what they would tell a younger brother about being kind, Sammy in the high prosocial group responded, “I’d tell him to treat people the way you want to be treated. Don’t be easily provoked…Sometimes they make take your kindness as a weakness, but I feel like I should just treat people like you want to be treated.” In response to this, Kenny stated, “Be kind, but don’t be too kind,” and Malcolm added, “Don’t be vulnerable.” This began an exchange in which they discussed how they may be taken advantage of if they are too kind to those around them. Kenny and Jared used a hypothetical situation to illustrate their point.

Kenny: Let’s say I come—we come to school every day and I give you a dollar every day. You be like thank you, thank you the first time I give you a dollar. If I constantly keep giving you that dollar you gonna expect me to did not giving you that dollar.

Jared: Then that one day you don’t give them a dollar!

Kenny: Then they are gonna get mad at you, ‘Where’s my dollar?!’ I’m like ‘Yo dollar?! What you mean?! I gave it out my heart, my kindness.

Kenny explained one consequence of their oppressed status, saying, “It’s being vulnerable because they feel like us being Black, we’re vulnerable to rap. They feel like if they provoke us enough, we’ll explode.” They went on to explain their identities as Black males made them more susceptible to being taken advantage of or unfairly tested. Malcolm stated, “The fact that we’re Black men, we’re the target anyways. They’re going to try to see if the stereotypes if they hear are true…Some folks in the world—they want to see you—I don’t know—they want to see you fail.”

Participants stated there are only a few people with whom they can be vulnerable and kind. They named their mother as one with whom they were unabashedly open and generous. As one participant stated, "My mom, that's all I need in this world." They went on to state certain contexts afforded vulnerability such as a sports game or graduation, but generally, they were very protective of their feelings, which influenced the ways they engaged with others.

## DISCUSSION

This study examined adolescent Black males’ talk about their racially gendered identity and prosocial behaviors, the relationship of these constructs to each other, and the role of systemic bias in the meaning‐making process. Theory (Crenshaw, 1991; Spencer, 2006, 2008) and evidence (e.g., Belgrave et al., 2011) prompted us to direct our investigation to Black boys specifically; their intersectional social positioning contributes in particular ways to their development. The participants were divided into two focus groups: highest reported prosocial behaviors and lowest reported prosocial behaviors as determined by scores on self‐report surveys in a previous study. Qualitative results revealed how the groups discussed their identities, dehumanization, prosociality, and the meaning‐making they constructed about the intersection of these aspects of their lives.

First, across groups, participants reported feeling positive about their identity. Second, both groups of boys spoke about their social positioning and the low regard others have for them as Black males. Participants in the low prosocial group were more likely to identify this dehumanization as arising from the hostility of Whites. Third, despite or because of living in a world that devalues them, these boys claimed the greatest value in interpersonal relationships is respect and, further, that acts of kindness are part of their racial identity. Fourth, participants made meaning by articulating an interaction between their social context, identity, and prosociality. They stated negative stereotypes and poor treatment of Black males motivated them to exhibit acts of kindness to “prove them wrong.” The high prosocial group further argued that it is important to not be too kind to others, for fear of appearing vulnerable and open to provocation by others. The outstanding difference between the high and low groups can be seen in their conceptualization of their social context. The low prosocial group viewed the outside world as full of racialized danger from a named group. The high prosocial group declined to detect danger based on race alone, but nonetheless felt their prosociality put them at risk of being viewed as a vulnerable target.

### Emergent Identities in the Context of Dehumanization

Despite participants’ awareness of others’ negative appraisals of them, they were positive about their racially gendered identities, mirroring earlier quantitative findings (Harris & Kruger, 2019). Interestingly, participants could not specify feelings about their gender identity as easily as they could about their REI. However, when asked which identity was more salient, they claimed to be speaking from their positions as Black males, asserting “they come together.” As an intersectionality framework asserts, socially mediated identities do not function in parallel nor are they merely additive, because they are neither independent nor neutral in their construction. They are inherently relational and the result of systemic and structural inequity. Participants easily talked about their shared experiences as Black males and acknowledged the inextricability of race and gender in their lives. However, they spontaneously attributed their experiences to race. This may reflect internalized beliefs that social identities operate in parallel as well as the unique roles race and racism play in their experiences.

Participants acknowledged inherent risks in being young Black males in America. Indeed, they used words such as “feared” and “targeted” to describe how others view them, demonstrating the existential fear that adolescent Black males must carry. Reflected appraisals (a person’s perceptions of others’ evaluations of them) shape emergent identities (Spencer, 2008). As predicted by P‐VEST, the hostile environment of anti‐Blackness (and anti‐Black maleness) was threaded through their thinking. These teenage boys spoke about the tension between their own regard for themselves and the low regard they believed others have for them. They noted that even “positive” stereotypes of adolescent Black males (e.g., athletic ability) relegated them to a subordinate position. They actively grappled with how to define themselves in ways that did not affirm oppressive forces and did represent their full selves.

Scholars have noted that Black males must develop an identity in a terrain in which they are simultaneously desired and despised (Cooper, 2013; Stevenson, 1997). As Boykin and W.E.B. Dubois point out, Black people must often create a “double consciousness” to survive within America (Boykin, 2020; Du Bois, 1994). Participants’ responses suggest many are in the process of building this very consciousness as it relates to their emergent identities. As noted by one participant, these tensions changed and became more apparent as he “got bigger.” He was aware that society’s perception of him became more negative as he entered adolescence, reflecting previous research (Goff et al., 2014). This suggests, at least for these Black males, the creation of a double consciousness does not begin in emerging adulthood but rather in adolescence.

A difference between the focus groups emerged in the participants’ interpretation of the forces at play that create hostility and discrimination in their environment. As found previously in the quantitative study, those participants who scored lower on a measure of prosociality also scored lower on racial public regard, or how they believe others see them. In their focus group the low prosocial participants recounted episodes of being treated negatively because of their social position, and they used “othering” language in their narratives, referring to those who mistreated them as “they.” When probed, the participants described “they” as Whites and painted a picture of their social world as a battlefield where White and Black Americans are continually at odds. The low level of prosocial behaviors they reported in their immediate social network could be a coping response to their perceived powerlessness in the face of discrimination. They also may be unwittingly incorporating society’s negative stereotypes about Black males into their burgeoning identities (Stevenson, 1997). Participants in the high prosocial group described similar experiences of discrimination. However, when probed, they declined to assign a race to the perpetrators of discrimination and claimed race should not be a factor in interpreting another’s behaviors. The high prosocial group acknowledged hostility in their environment but attributed it to the character rather than the color of others. In line with the anti‐Blackness framework, this could be because participants who have been socialized to be prosocial may also have been socialized to avoid naming the explicit ways racism, intentional or not, shapes their daily interactions.

### Prosociality at the Nexus of Identity and Context

Participants’ conceptualization and evaluation of kindness and respect revealed macro‐level environmental influences on their thinking. For example, the participants understood prosociality as a cultural value of the Black community and a means to community cohesion (“It’s just the way we were brought up”). These findings recall previous studies illustrating the contribution of racial socialization messages to prosociality (Lozada et al., 2017; Mayia et al., 2021; White‐Johnson, 2012, 2015).

In both focus groups participants emphasized respect and suggested respect encouraged them to engage in prosocial behaviors. Their emphasis on respect may arise from daily experiences of disrespect across contexts. Adolescent Black males’ identities develop in an environment that often “disrespects” their independence and individuality (Stevenson, 1997). Their perceived inability to safely respond to disrespect from macro‐level sources (e.g., media and policies) and authority figures (police and teachers) could be a reason why they see respect as an essential feature of relationships. Moreover, their helplessness in the face of higher authorities and systems may be why adolescent Black males respond in violent, anti‐social ways against peers when they feel disrespected.

When asked to elaborate on respect, participants provided examples of deference. This reflects the cultural value of deference to elders often found in Black communities (Harvey & Rauch, 1997). Participants’ examples of deferring to teachers demonstrates the enactment of this value. However, it also may reflect the internalization of systemic oppression that forces Black males to submit. Research has documented how oppression engenders passivity in Black bodies. For example, in schools serving predominately Black communities, obedience is valued over individual agency (Sondel et al., 2019). Indeed, deference can be a matter of life and death for adolescent Black males. In 2012, when the late Jordan Davis, a 17‐year‐old adolescent Black male, did not defer to the wishes of Michael Dunn to lower the volume of his music, Dunn shot him 10 times (Cheng, 2018). Moreover, police cite “not following instructions” as justification for the murder of young Black men (Smiley & Fakunle, 2016). The macro‐environment also contributes to the ways prosociality is enacted. For example, the high prosocial group contended they had to limit their acts of kindness to avoid appearing vulnerable to those around them, arguing their social positioning causes people to “test” them. These participants may have been describing the “cool pose,” high levels of bravado combined with apathy and disengagement, enacted as protection from the social and/or physical repercussions that may occur in its absence (Cunningham & Meunier, 2004; Jackson, 2018). A danger arises when these behaviors are performed *across* contexts, and the “cool pose” is interpreted as arrogance or aggression, as happens in schools. Cassidy and Stevenson (2005) point out that these hyper‐masculine performances leave Black males in a hyper‐vulnerable position. Despite enacting disengagement, adolescent Black males often struggle with their real need for emotional connection (Rogers & Way, 2018). Participants’ emphasis on family for emotional support reflects prior research that demonstrates Black males often turn to close family members to process the oppression they experience (Jackson, 2018). Participants also noted that vulnerability and prosociality were safe for them to express with family, another example of the power of the social context to affect the enactment of prosocial behaviors.

### Meaning Making

The interaction of factors in these adolescent Black boys’ social development is illustrated in these findings. The participants described the complexities and contradictions they negotiate in constructing a self and moving through the world. For example, the participants feel pride about who they are and at the same time understand that the larger world is hostile because of who they are. They describe how their identity includes the values of kindness and respect, while others stereotype them as inherently callous and dangerous. To protect their identity and possibly their physical integrity, these youth go out of their way to be prosocial, to dispel negative stereotypes. At the same time, they guard their behavior and try not to be *too* prosocial, lest they appear vulnerable and liable to be tested. They understand themselves as misunderstood by others, and they must monitor their behavior in real time (engage prosocially; don’t engage) to protect themselves.

These findings echo established predictors of prosociality in adolescent Black boys (e.g., Lozada et al., 2017). The high prosocial group in the present study reported experiences related to the predictors of REI, discrimination, identity as oppressed, and parental racial socialization. Like others, we also found prosociality was motivated to dispel stereotypes (Mayia et al., 2021) and to contribute to the community (White‐Johnson, 2012, 2015). We originally conceptualized prosocial behaviors as a stage specific outcome, but they can also be seen here as reactive coping mechanisms. In both groups, participants stated they engaged in prosocial actions despite *or* because of the low regard others have for them. One may not think of “helping behaviors” as a coping mechanism but where adolescent Black males are considered threatening, prosociality creates a counternarrative for others and possibly also for themselves.

Our findings align with these previous ones but go beyond them to illustrate the risks of prosociality that adolescent Black boys must weigh explicitly because of their social positioning. Racial‐ethnic (or racially gendered) identity in Black boys can promote prosociality (to cope with and rebut stereotypes) and community engagement (as an act of solidarity), but also can promote guardedness and withdrawal from engagement (to look imperturbable and deflect threat). The development of prosociality is frequently characterized in relation to micro‐level influences like peers and parents, but these findings indicate the macro‐level context of racial discrimination and inequity influences how adolescent Black males think about the social world and the meaning and purpose of prosociality. Our participants’ beliefs about the fundamental elements of social relationships were constructed through their understanding of themselves as Black males which was, in turn, informed by anti‐Blackness and systemic oppression within the United States.

### Limitations and Future Directions

While this study uncovered valuable information about Black males’ social development, some limitations should be noted. Our small sample size and single location limit generalizability but allowed us to learn important detail about the participants’ thinking. We also were not able to look at differences by age or over time. A longitudinal study may reveal shifts in these processes. Additionally, grouping participants by prosocial scores may have led to the absence of more varied voices and exaggerated the group differences. Further, we realize social acceptability may have influenced participants’ responses in the focus groups. Last, we acknowledge that our data are based on boys’ quiet reflection in a safe space, and that may be different from their behavior in real time. Despite these limitations, this study demonstrates the value of using qualitative approaches, the P‐VEST framework, and an anti‐Blackness lens to understand pathways and outcomes for youth of color.

One finding meriting future investigation was the importance of respect to these adolescents’ thinking about relationships. Future examination of the function and development of respect can elucidate its relationship to adaptive developmental outcomes. Similarly, reflected appraisals, particularly the influence of low racial public regard, were central to our participants’ thinking and merit further investigation to explicate the processes of development in a hostile context.

These findings also demonstrated the ways adolescent Black males both accommodate and resist societal expectations of them. Future research should explore the risks and benefits for development that arise as marginalized youth manage dominant messages about who they should be and how they should act. Moreover, given the specific experiences attached to racially gendered identity in these findings, using an intersectional lens will be essential to further our understanding.

Finally, as previously noted, adolescent Black males must negotiate tensions between their private regard and public disregard during their adolescence *because* of their entrance into adolescence. Thus, future research on young Black males must include a developmental perspective that explicitly considers the role of macro‐levels processes like racism and discrimination in normative developmental outcomes.

## Footnotes

^1^
Donald J. Trump was the president of the United States (2016–2020) when these focus groups took place.

## APPENDIX A

### FOCUS GROUP QUESTIONS

1. I want to learn about what your experiences as young Black men.
  - Can we start off today’s conversation by you guys sharing with me your experiences as a young Black male attending an urban charter high‐school and the positives and negatives that you guys may experience?
2. Now I want to keep talking about your how you may respond to situations, but shift gears a little bit and talk about being kind to others.
  - How do you define being kind to another person? How about another person showing kindness?
  - Can you think back over the past couple months and share a time you showed kindness to another person?
  - How do people’s feelings about your race influence your treatment of them?
3. Let’s keep thinking about being young Black men in America. I want to ask each of you to think for a minute about 3–4 words that come to mind when you think of yourself as a Black person.
  - How do these feelings about your race affect how you engage with others?
4. Now, a slightly different question, what 3–4 words that come to mind when you think of yourself as a male.
  - How do these feelings about your gender affect how you engage with others?
  - And as a Black male?
5. I want to learn more about your perceptions of other people’s views of your race and gender. Can you guys share with me 3–4 words that you think come to ***other people’s*** mind when they see young Black males?
6. Can you think back over the past year and tell me about experiences where you feel you have been negatively viewed and/or treated because of your race?
  - How did you respond to these experiences?
7. Now a slightly different question, can you share a time where you feel you have been *viewed/treated positively*?
8. How did you respond to this experience?
9. Can you think back over the past year and tell me about experiences where you feel you have been negatively viewed and/or treated because of your gender?
  - How did you respond to these experiences?
10. Now a slightly different question, can you share a time where you feel you have been *viewed/treated positively*?
  - How did you respond to this experience?

## APPENDIX B

### PREVIOUS STUDY MEASURES

Quantitative results from the previous study informed the procedure and analysis of the present study. The following scales were used to measure the racial identity, gender identity, and prosocial behaviors. The Multidimensional Inventory of Black Identity‐Teen (MIBI‐T) measured racial‐ethnic identity (Scottham et al., 2008) This study used the centrality and regard (private and public) scales. The centrality measure examines how central race is to a person’s identity. The private regard measure examines how the person feels about being Black and the public regard measure (α = .80) investigates how the person feels others think about Black people. Higher scores for regard indicate more positive public and private regard and higher centrality score indicates higher levels of centrality.

In line with prior research on adolescent Black males’ racially gendered identities (Rogers et al., 2015) this study adapted items from the MIBI‐T to examine gender identity. Participants’ gender centrality and gender regard were examined. *Male* replaced the word *Black* on each item on the three measures. For example, a centrality item was adjusted to “I feel close to other males.”

The study used the Prosocial Behavior Scale, created by the Developmental Studies Center (Solomon et al., 2000). The nine‐item scale measures participants’ frequency of prosocial behaviors on a 5‐point frequency scale (1 = *never* to 5 = *more than 10 times*) in the last semester. Higher scores indicate higher engagement in prosocial behaviors. Sample items include “Helped your classmate with homework” and “Tried hard not to hurt someone’s feelings.”
